# Supplementary material for: Understanding public trust in information about interim nuclear waste storage: The roles of acceptance, gender, and proximity
Source: PLoS One. 2025 May 29;20(5):e0323896. doi: 10.1371/journal.pone.0323896 (PMC12122037; doi:10.1371/journal.pone.0323896)
Supplement: Attachment — Submitted filename: Review for Manuscript Number PONE-D--24-58280.docx [file pone.0323896.s002.docx]

**Review for Manuscript Number PONE-D-24-58280
"Understanding public trust in information about interim nuclear waste storage: The roles of acceptance, gender, and proximity**

Dear Editor,

I declare no conflict of interest in carrying out this review.

I will first summarize the research and provide my overall impression, then I will provide evidence and examples.

The authors have endeavoured to investigate the relationship between public trust in public authorities responsible for nuclear waste storage by considering the impacts of acceptance, gender and proximity. A survey was conducted among 1,558 former residents of Tomioka, Okuma, and Futuba, who were 18 years or older at the time of the Fukushima Daiichi nuclear power plant accident. Given the time lapse since the accident, most survey respondents were generally older than 65 years. Residents from these towns have taken part in previous research studies employing surveys in the past. The survey aimed to determine participants' trust in the information provided by public authorities regarding the interim storage facility. Generally, the authors found that men and younger persons exhibited higher levels of trust, and overall, 60% of study participants showed trust. Two distinct logistic regression models were employed to prevent potential bias from a single variable dominating the regression analysis. Specifically, Model I considered concerns of genetic risk, while model II considered negative image concerns. Concerns about genetic risks and negative images significantly decrease trust in both models. Good mental health significantly increases trust in both models. The methodology undertaken by the authors follows earlier published research from their team. The results generally support the conclusions of the study, however, several points of clarification are raised.

Specific comments are provided below for the authors consideration.

**Comment 1:** There is a great deal of literature grounded in psychology on trust, and the factors that influence trust. It is unclear whether the survey included a definition of trust so that all respondents had the same interpretation. The literature (for a meta-analysis see doi: [10.3389/fpsyg.2023.1081086](https://doi.org/10.3389/fpsyg.2023.1081086)) explains predictive factors of trustworthiness as an outcome (rather than asking about trustworthiness as a direct question). Important predictive factors include reputation of the trustee and the closeness of relationship between trustor and trustee. A description should be added to the manuscript as to what is meant by “factors”, how those “factors” were chosen, and what supporting literature is aligned with the (somewhat limited) choice of “factors”. Other research indicates that trust can be broken down into several factors: humanity, capability, transparency, and reliability (or a variation of these). For this reason, survey questions (to evaluate trust) should be framed as “was the information reliable/transparent?”, “do you think the authority in question is capable/competent?”, etc.

**Comment 2:** For completeness, consider adding the survey as a stand-alone electronic attachment to the article.

**Comment 3:** In the Discussion, one could challenge that your “analysis confirms that trust in information about radioactive waste and its storage depends significantly on the public acceptance of facilities”. Rather, does public acceptance depend on trust? Thus, do the questions you are posing in the survey lead to the appropriate conclusions? Or should the conclusion be reframed to discuss how trust is first established (and discuss how this occurred through outreach and engagement activities), followed by acceptance.

**Comment 4:** The concept of risk perception is explored but ill-defined. Suggest defining risk perception more clearly, including how risk perception was measured. The relationship between a greater level of trust and a lower risk perception is vague and should be qualified. Is risk perception simply the individual’s belief that genetic risks due to radiation exposure may or may not occur and general concerns about negative perceptions? The way in which individual’s formulate their personal risk perception, or how they judge a risk, is influenced by many factors that are not discussed or accounted for, including familiarity with the risk, understanding of the risk, uncertainty surrounding the risk, not to mention culture and worldview, among other important factors that should be either added to the discussion or mentioned in the limitations, separate from factors that impact trust.

**Comment 5:** The reference of Hunt and Frewer (1999), while groundbreaking at the time, is now 25 years out of date. Do the conclusions (perceived truthfulness/message bias and expertise/degree of knowledge) hold true today? How do they compare to the current literature published by social scientists touching on ionizing radiation. For example, newer research is focused on reducing the importance on knowledge and focuses instead on heuristic predictors (e.g., DOI: 10.1097/HP.0b013e31823fb5a5).

**Comment 6:** Also in the Discussion, the tone could be framed more positively with regards to mental health. As written the Discussion left the impression that people who had less stable mental health struggled to trust information or accept a facility. The fundamental issue is not resilience of the community (or the individual), but whether the authority deserves trust (are they capable, transparent, reliable, do they demonstrate humanity). Individuals with less stable mental health can still make that determination if presented with information. Further, of the survey population, were the woman those with reported lower mental health? If so, please comment on how this variable may be impacting your conclusions.

Thank you for the invitation to peer-review the manuscript.

Please reach out directly to me should you have any additional questions, or comments, or points of clarification.

Kind Regards.
